# Supplementary material for: Structural insights into isoform-specific RAS-PI3Kα interactions and the role of RAS in PI3Kα activation
Source: Nat Commun. 2025 Jan 9;16:525. doi: 10.1038/s41467-024-55766-x (PMC11718114; doi:10.1038/s41467-024-55766-x)
Supplement: Supplementary file 1 — Supplementary Information [file 41467_2024_55766_MOESM1_ESM.pdf]

Structural Insights into Isoform-Specific RAS-PI3Kα Interactions and the Role of RAS in PI3Kα Activation

Daniel Czyzyk[1#], Wupeng Yan[1,#+], Simon Messing[1], William Gillette[1], Takashi Tsuji[2], Mitsuhiro Yamaguchi[2], Shinji Furuzono[3], David M. Turner[1], Dominic Esposito[1], Dwight V. Nissley[1], Frank McCormick[1,4], and Dhirendra K. Simanshu[1*]

Supplementary Material

Supplementary Figures 1 – 19

Supplementary Tables 1 – 3

Scheme 1

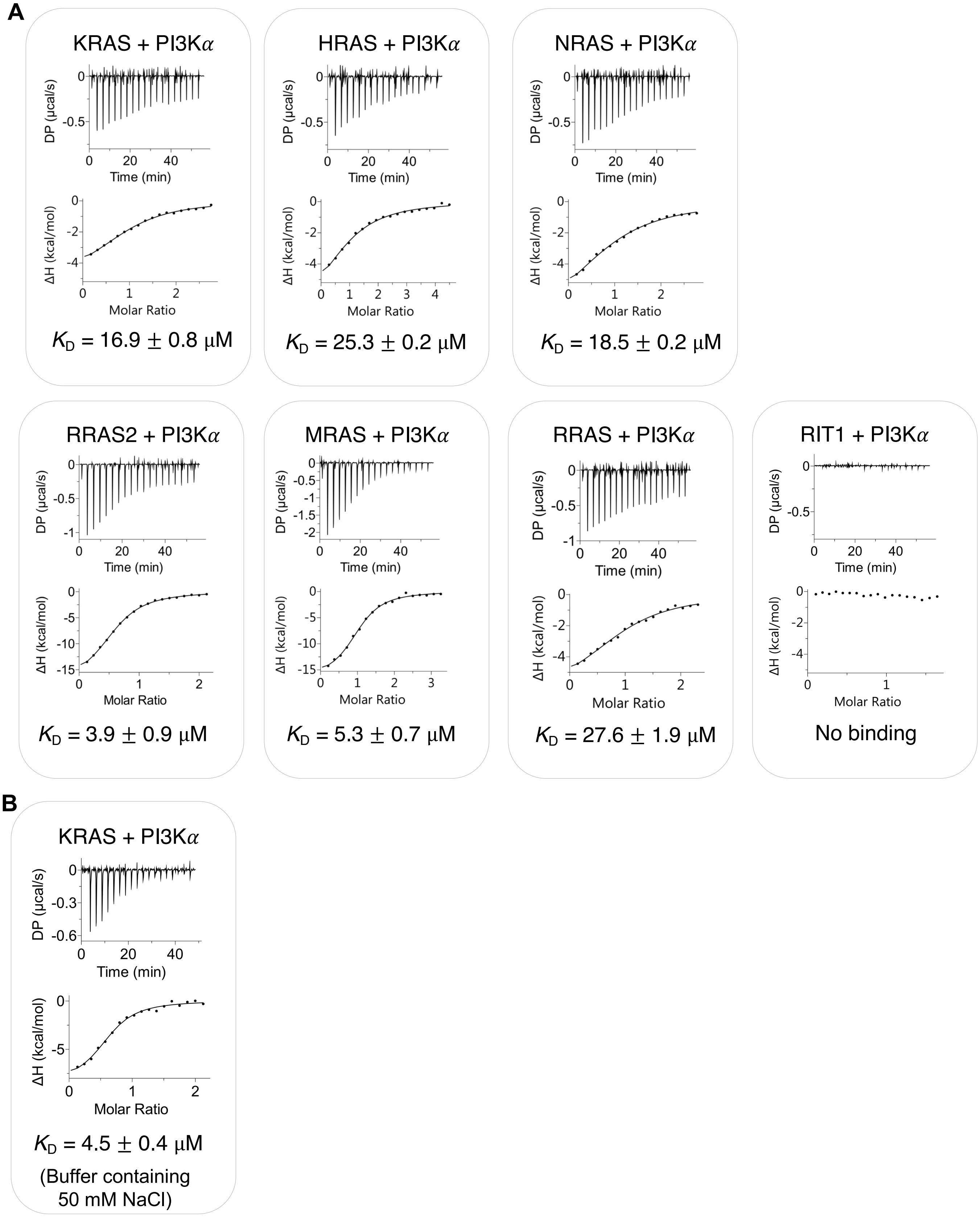

Supplementary Figure 1: Assessing the binding affinity between RAS subfamily members and PI3Kα. **(A)** ITC experiments were conducted under physiological conditions (150 mM NaCl, pH 7.4) to determine the dissociation constant (K_D) for GMPPNP-bound RAS proteins (KRAS, HRAS, NRAS, RRAS, RRAS2, MRAS, and RIT1) binding to PI3Kα. **(B)** Reducing the salt concentration from 150 mM to 50 mM NaCl enhanced KRAS binding to PI3Kα, lowering the K_D from 16.9 µM to 4.5 µM. K_D values are presented as mean ± standard deviation from two technical replicates. The differential power (DP) represents the heat differences measured between the reference and sample cells during titration.

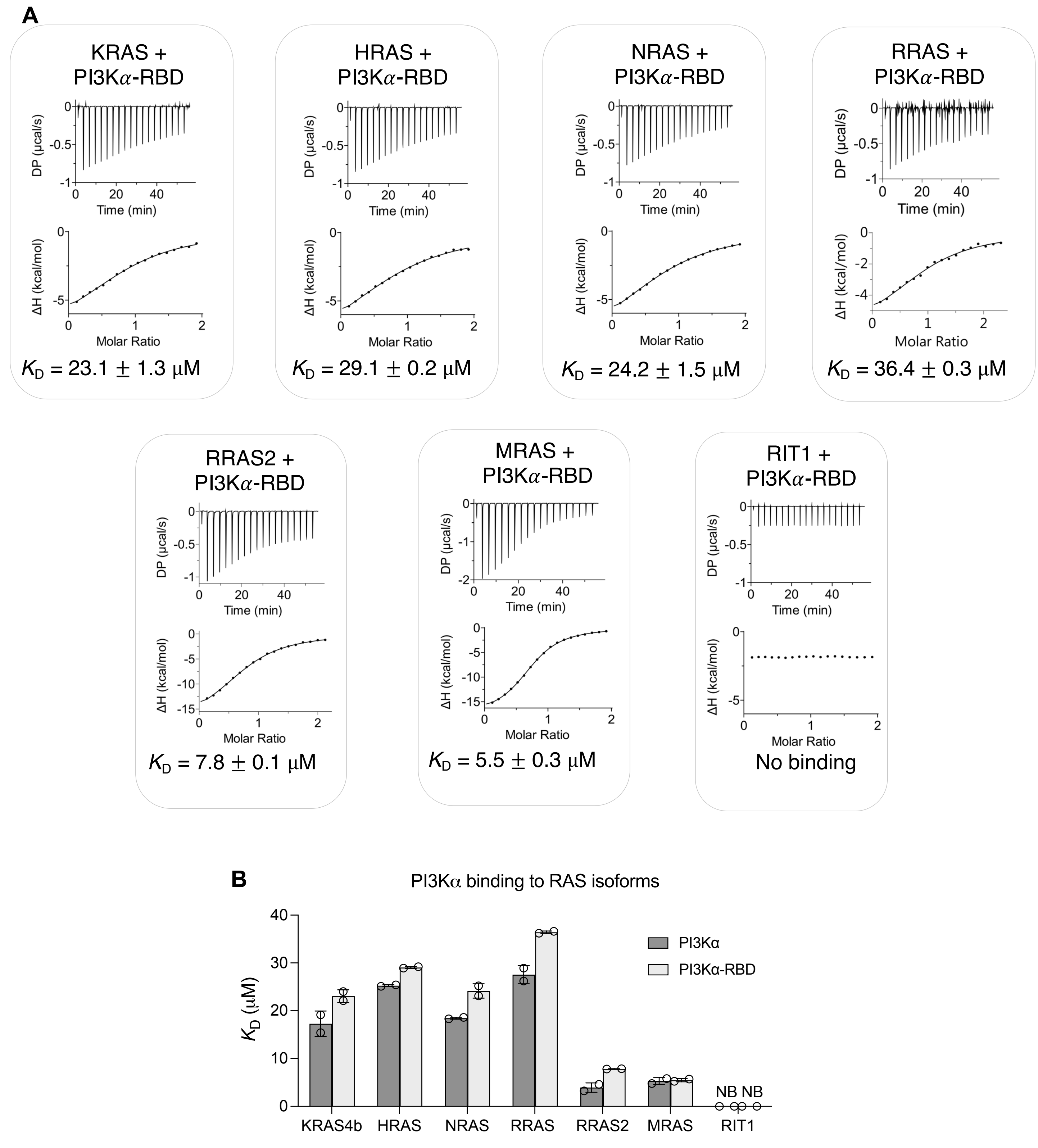

Supplementary Figure 2: Comparison of binding affinity of RAS subfamily members with PI3Kα vs. PI3Kα-RBD. (**A**) ITC experiments were performed to determine the dissociation constant (K_D) between PI3Kα-RBD and various RAS family proteins (GMPPNP-bound), namely KRAS, HRAS, NRAS, RRAS, RRAS2, MRAS, and RIT1. K_D values are presented as the mean ± standard deviation derived from two replicates. (**B)** A bar graph showing the dissociation constant (K_D) of PI3Kα and PI3Kα-RBD with RAS family members, as presented in Supplementary Figures 1A and 2A. The K_D values derived from PI3Kα-RBD are in close agreement with those from the PI3Kα (p110α + p85). The standard deviation is shown as error bars, with two replicates shown as circles. NB: no binding.

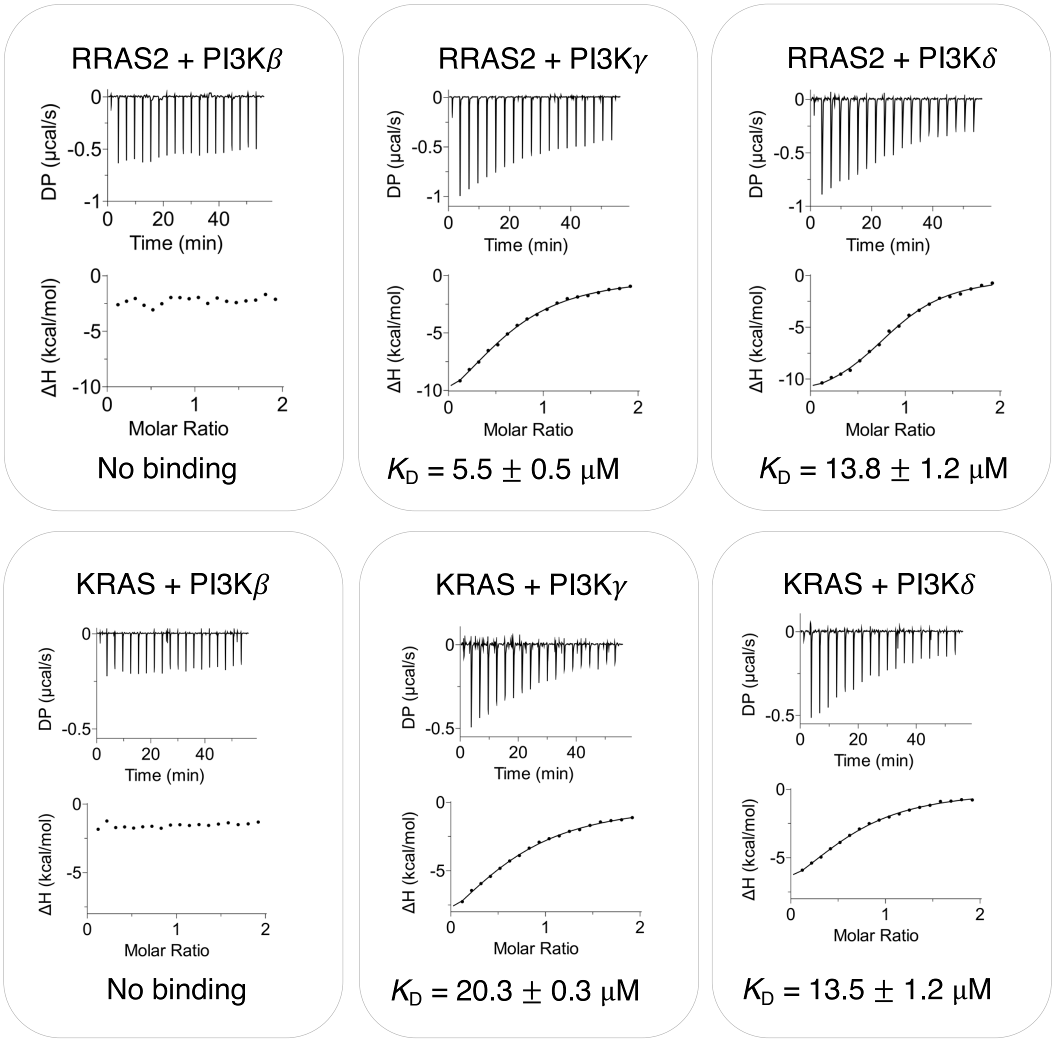

Supplementary Figure 3: Evaluating the binding affinity of KRAS and RRAS2 with class I PI3K isoforms. ITC measurement of the binding affinity of GMPPNP-bound KRAS and RRAS2 with four class I PI3K isoforms: PI3Kα, PI3Kβ, PI3Kγ, and PI3Kδ. ITC profiles showing the binding affinity of KRAS and RRAS2 to PI3Kα are shown in Supplementary Figure 1A. K_D values are presented as the mean ± standard deviation derived from two replicates.

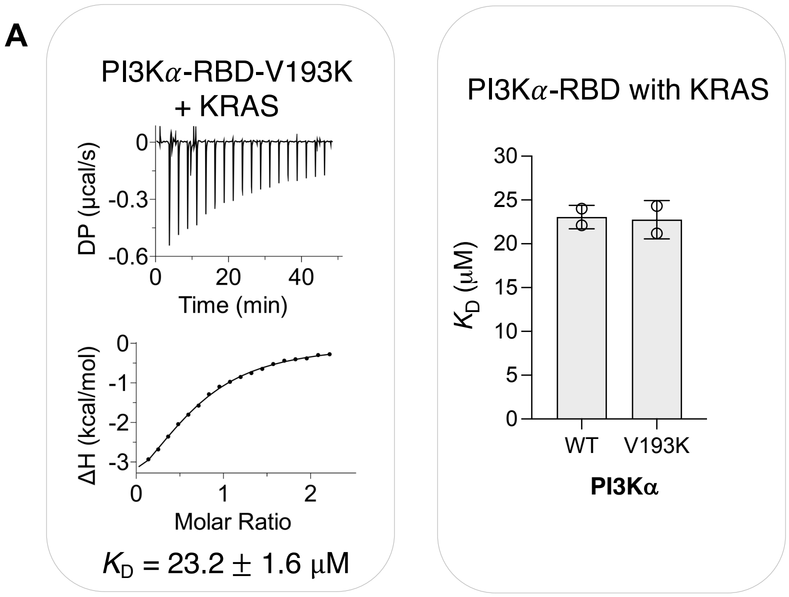

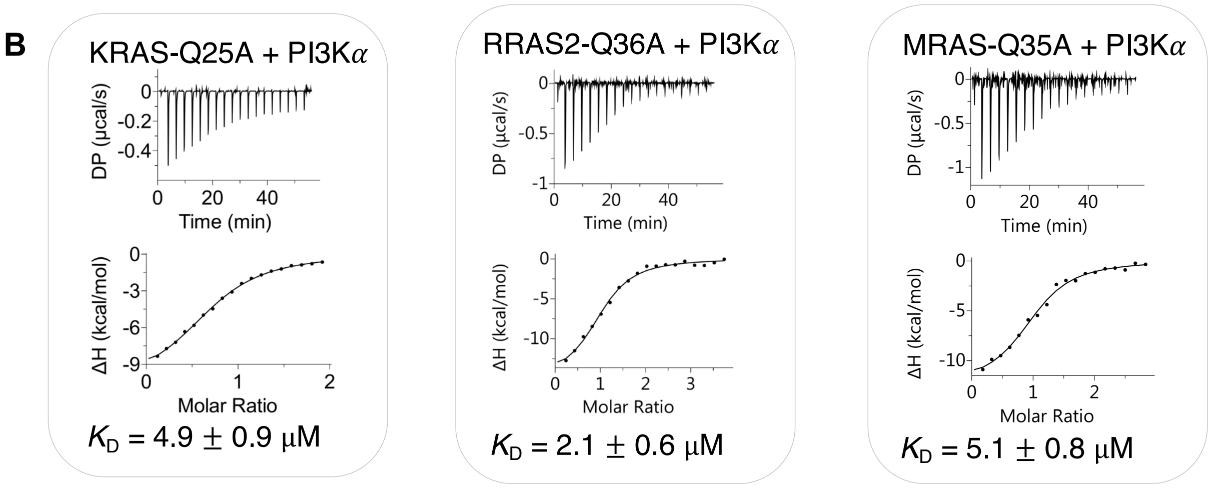

Supplementary Figure 4: Assessing the binding affinity of point mutants of PI3Kα and RAS for enhanced RAS-PI3Kα interaction: (A) Results from the ITC experiment measuring the binding affinity of KRAS (GMPPNP-bound) with the V193K mutant of PI3Kα-RBD (right). The bar graph in the left panel compares the binding affinity of wild-type (WT) and V193K mutant of PI3Kα-RBD, indicating no increased affinity with KRAS for the mutant. The standard deviation is shown as error bars, with two replicates shown as circles. (**B**) Results from the ITC experiments measuring the binding affinity of PI3Kα with the point mutants KRAS-Q25A, RRAS2-Q36A, and MRAS-Q35A (GMPPNP-bound). K_D values are presented as the mean ± standard deviation derived from two replicates.

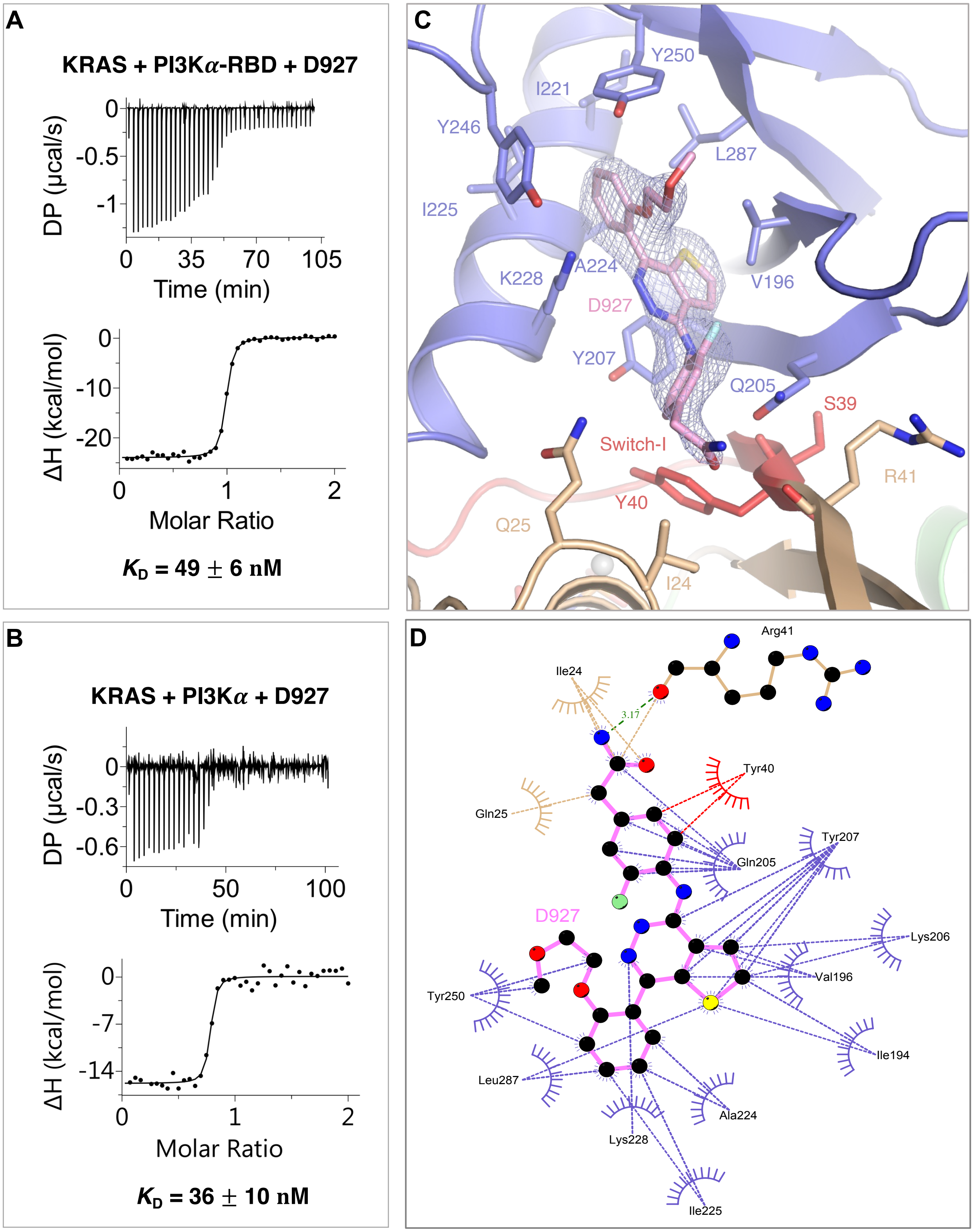

Supplementary Figure 5: Binding affinity and structural analysis of KRAS and PI3Kα Interaction with D927. (A, B) ITC measurements showing the binding affinity of KRAS (GMPPNP) with (A) PI3Kα-RBD and (B) PI3Kα in the presence of D927. K_D values are presented as the mean ± standard deviation derived from two replicates. **(C)** Omit map showing electron density corresponding to D927 within the PI3Kα-KRAS interface, contoured at 2.0 sigma. **(D)** Interactions formed by D927 (magenta) with various PI3Kα-RBD (blue) and KRAS (wheat and red) residues in the crystal structure. The dotted lines indicate interactions (less than 4 Å) of D927 atoms with various PI3Kα and KRAS residues. The schematic was generated using LigPlot+ v2.2.

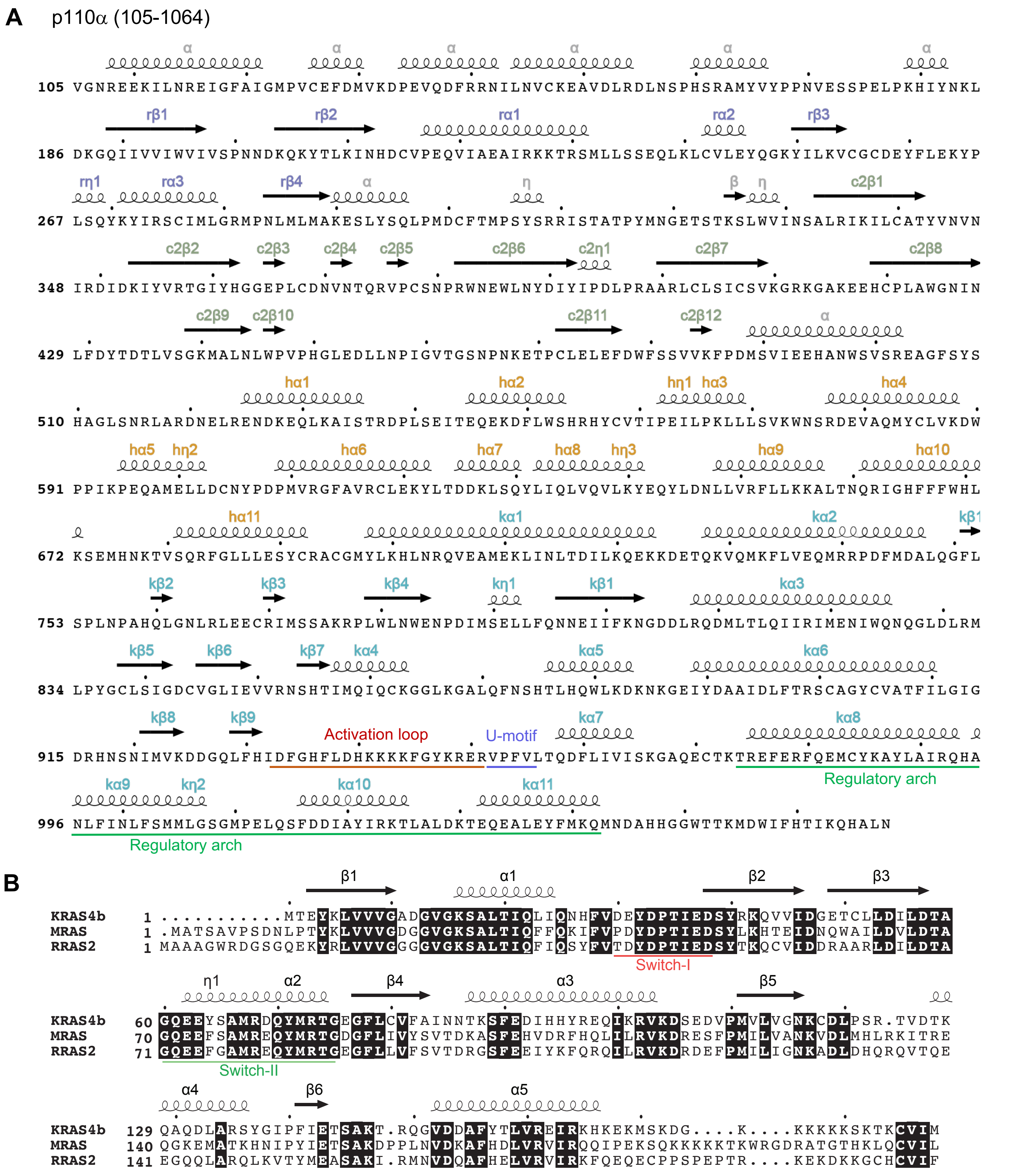

Supplementary Figure 6. Amino acid sequence analysis of p110α and RAS proteins with their secondary structural elements. (A) Amino acid sequence of the human p110α (residues 105-1068) protein used in the structural analysis of this study. Above the sequence, the secondary structure observed in the crystal structure is depicted, with helices represented as squiggles and strands as arrows. The secondary structural elements in various domains were numbered for each individual domains. **(B)** Amino acid sequence alignment of human KRAS4b, MRAS, and RRAS2 proteins. Totally conserved residues are shown in bold and highlighted in black. The secondary structural elements of KRAS4b are depicted above the aligned sequences.

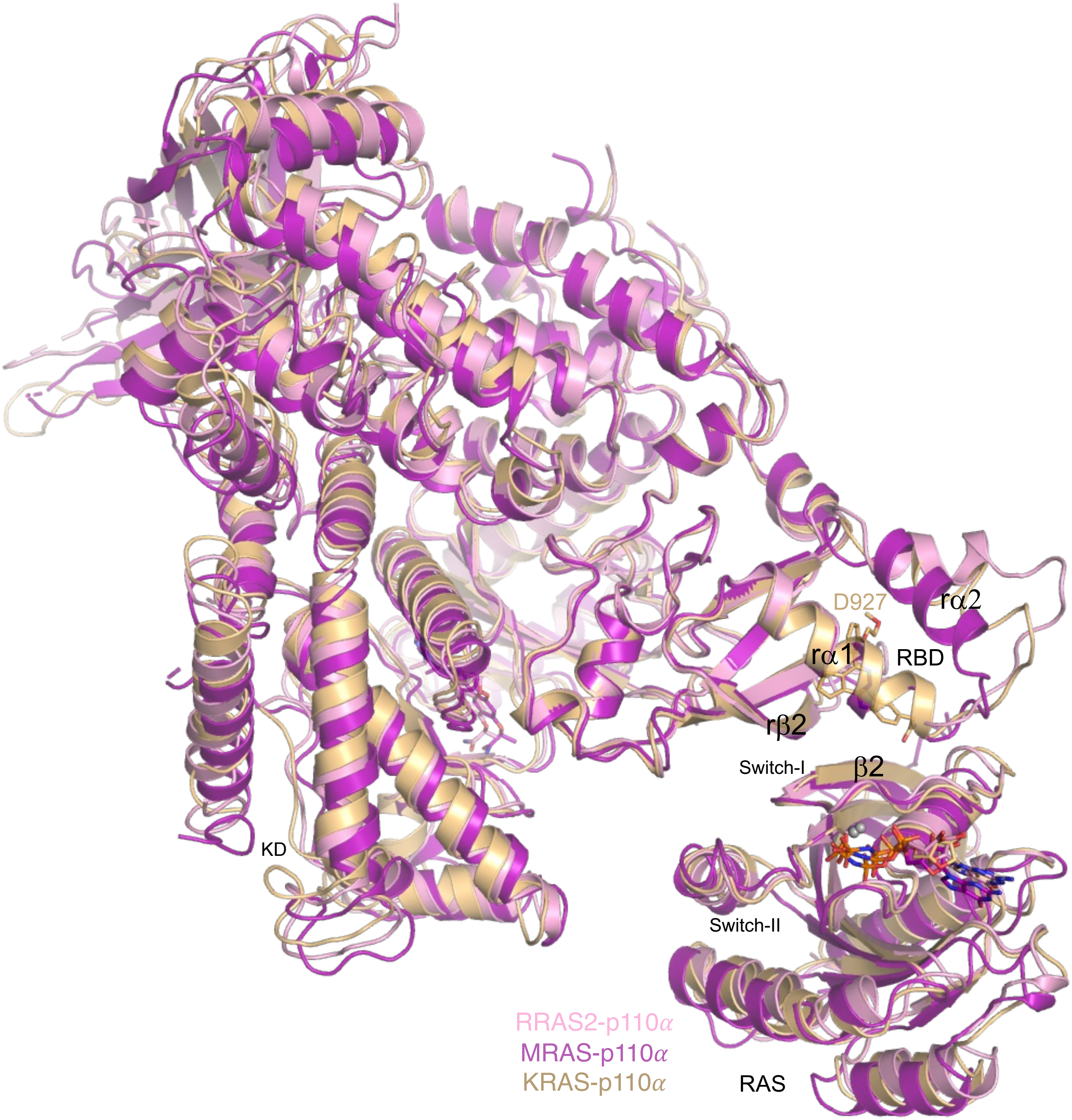

Supplementary Figure 7: Comparison of RAS-p110α complexes aligned using RBD to examine conformational changes in RAS and other domains of p110α. RRAS2, MRAS, and KRAS complexes with p110α are colored pink, purple, and wheat, respectively. The structural alignment reveals minimal conformational changes in the switch-I and β2-strand of RAS proteins and p110α-RBD, except for the rα1-rα2 loop and rα2 helix. Domains distant from the interaction interface exhibit minor rigid body movement in the p110α due to differing crystal contacts.

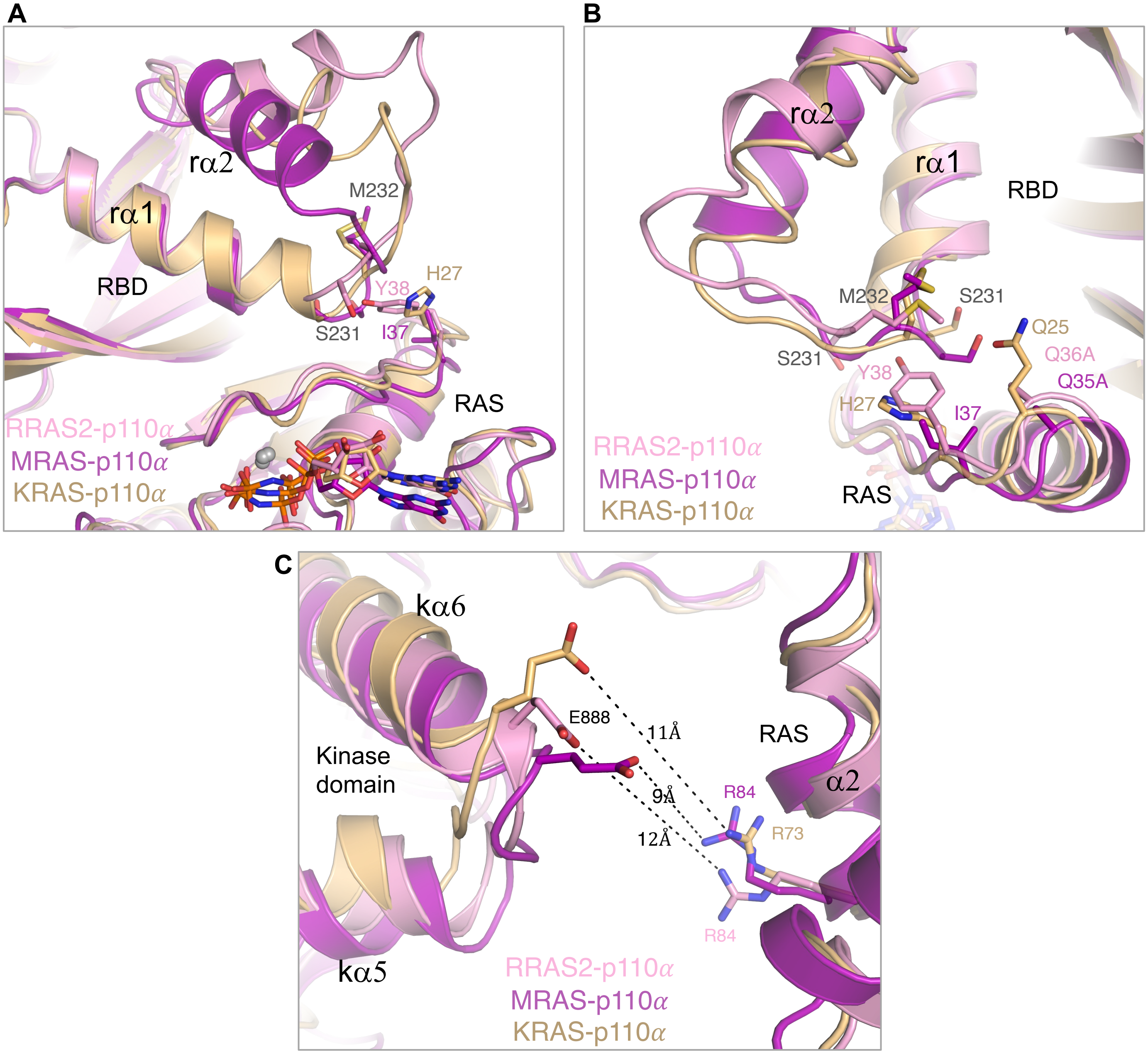

Supplementary Figure 8: Comparison of RAS-p110α complexes aligned using RBD to examine conformational changes at the interaction interface. RRAS2, MRAS, and KRAS complexes with p110α are colored pink, purple, and wheat, respectively. (A) Enlarged view of the RAS-p110α complexes highlighting the conformational changes in the rα1-rα2 loop and rα2 helix of the p110α-RBD due to compositional differences in RAS proteins at the interaction interface. Residues H27, I37, and Y38 of KRAS, MRAS, and RRAS2 interact differently with S231 and M232 present in the rα1-rα2 loop of p110α-RBD. **(B)** Enlarged view of the RAS-p110α complexes showing interactions formed by Q25, A35, and A36 in KRAS, MRAS, and RRAS2 at the interaction interface. These residues are adjacent to H27/I37/Y38 residues in KRAS/MRAS/RRAS2, forming unique interactions at the interface. **(C)** Enlarged view of the RAS-p110α complexes showing the distance (dotted line) between R73/R83/R84 in the switch-II region of KRAS/MRAS/RRAS2 and residue E888 in the C-lobe of the kinase domain of p110α. In the HRAS-PI3Kγ complex (PDB: 1HE8), the closest distance between these two residues is 4.5 Å.

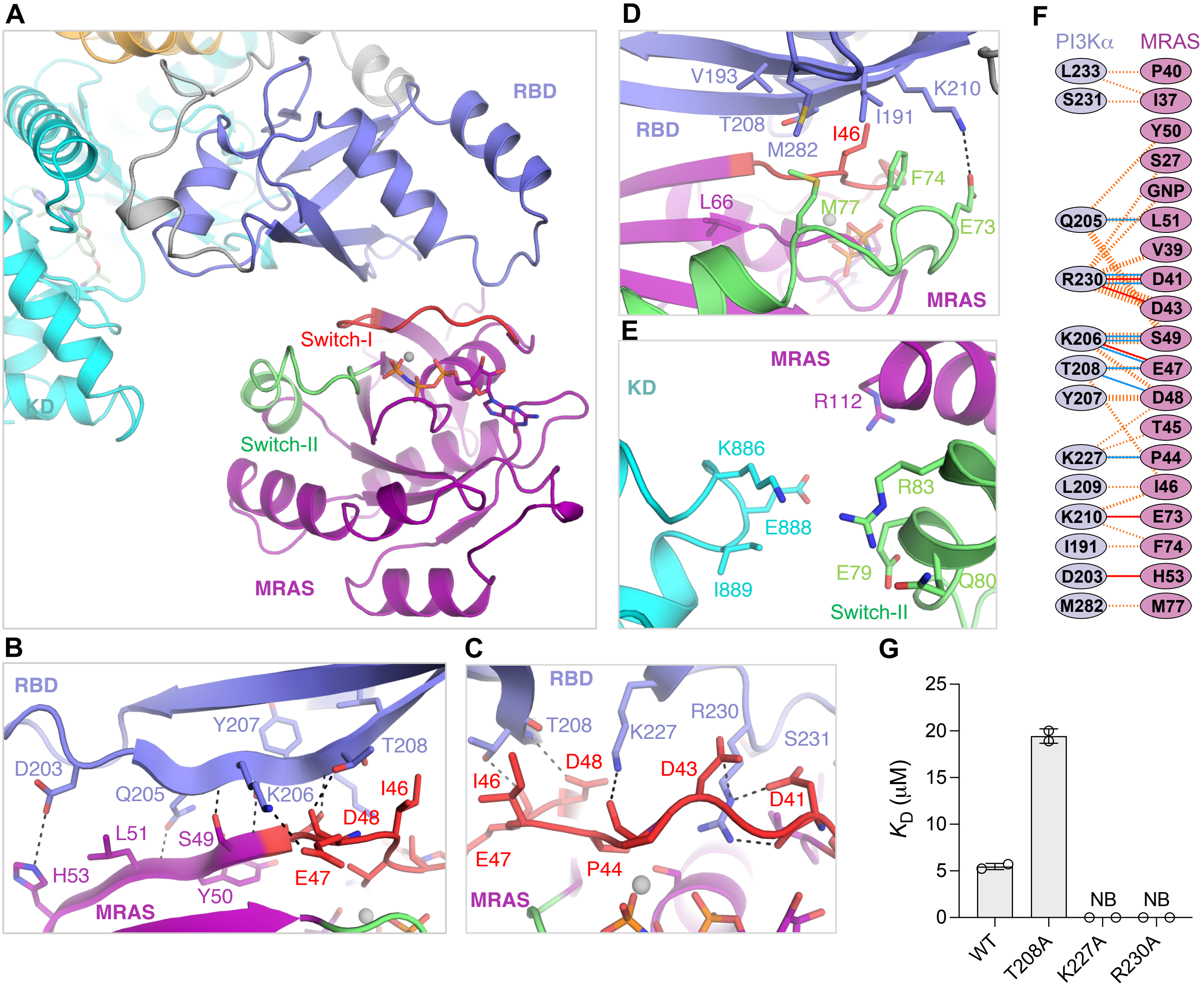

Supplementary Figure 9: Structural and mutational analysis of the MRAS-p110α interaction interface. **(A)** Enlarged view of the MRAS-p110α complex, highlighting the interaction interface. The domains of p110α and switch regions of MRAS are colored as in Figure 2D. **(B)** Interactions between the β2-strand of MRAS and the rβ2-strand of p110α. **(C)** Details of the interactions between the switch-I region of MRAS and the rα1-helix of the p110α RBD. **(D)** Interactions of the switch-II region of MRAS with the p110α RBD. **(E)** The interaction interface displaying residues on the C-lobe of the kinase domain facing towards residues on the Switch-II region of MRAS. **(F)** Schematic overview of the MRAS-p110α interaction interface made using the PDBSum server. Interactions are denoted by solid blue lines for hydrogen bonds, solid red lines for salt bridges, and striped-orange lines for non-bonded contacts (the width of the striped line is proportional to the number of atomic contacts). **(G)** Bar graph presenting the binding affinity (K_D) measured using ITC for point mutants of p110α residues at the interface with MRAS (GMPPNP). The standard deviation is shown as error bars, with two replicates shown as circles. NB: no binding.

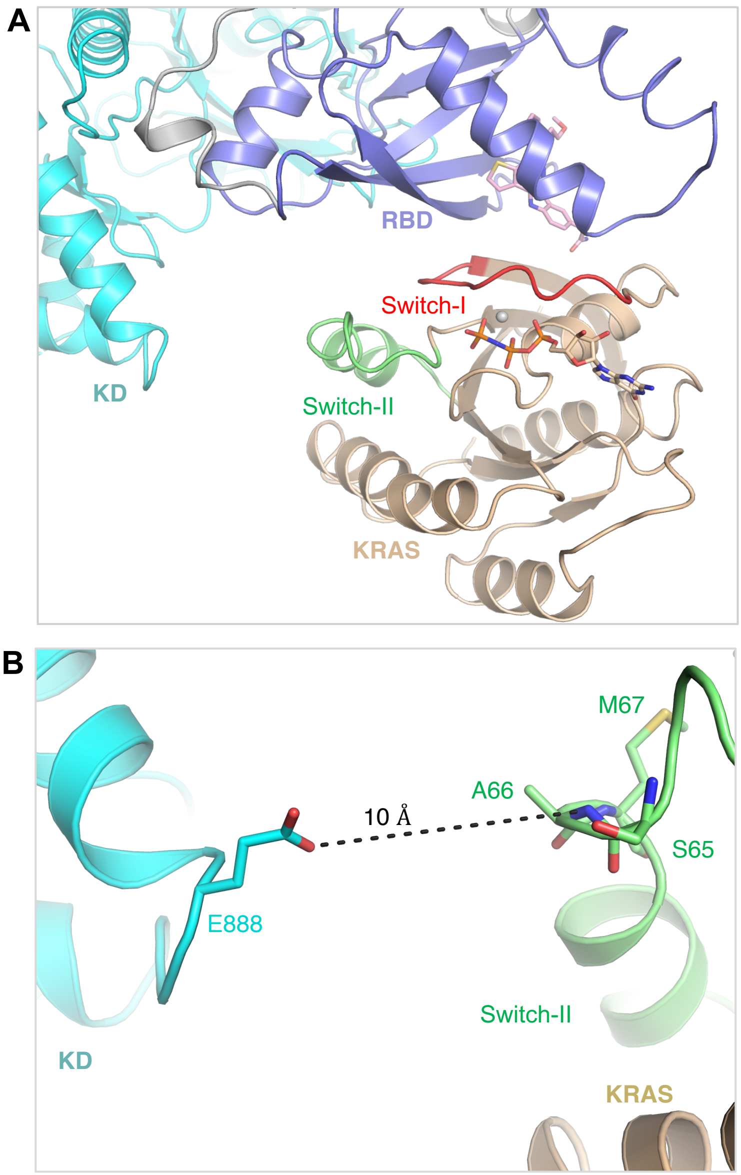

Supplementary Figure 10: Structural Analysis of the KRAS-p110α Interaction Interface. (A) Enlarged view of the KRAS-p110α complex, highlighting the interaction interface. The p110α domains and KRAS switch regions are colored as in Figure 2F. **(B)** Close-up of the interaction interface showing the switch-II region of KRAS and the C-lobe of the p110α kinase domain, with the shortest distance between two atoms from these regions indicated by a dotted line.

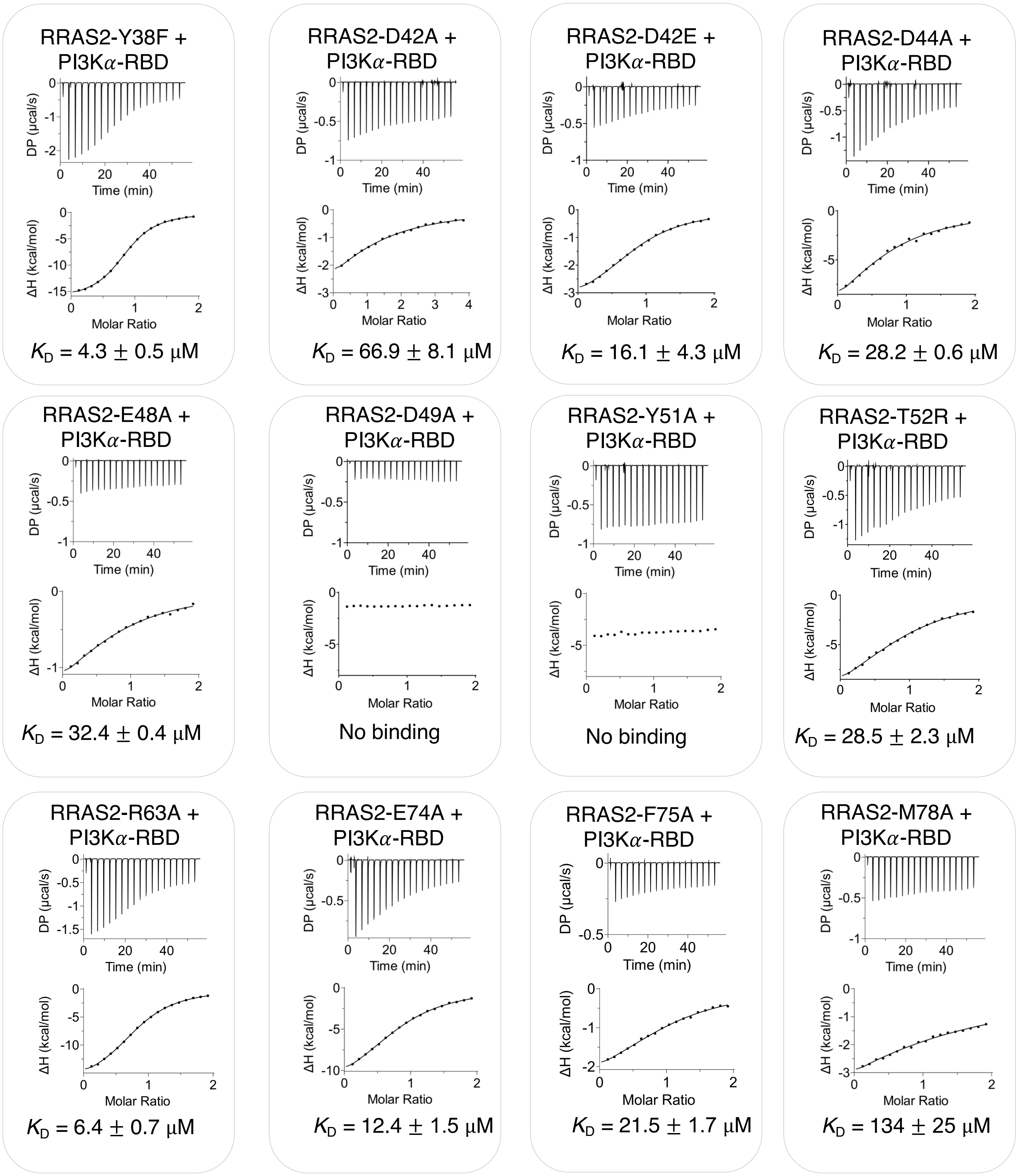

Supplementary Figure 11: ITC profiles showing the binding affinity of GMPPNP-bound RRAS2 mutants with PI3Kα-RBD. RRAS2 residues present near the interaction interface were mutated and examined using ITC to assess their impact on the RRAS2-PI3Kα interaction. K_D values are presented as the mean ± standard deviation derived from two replicates.

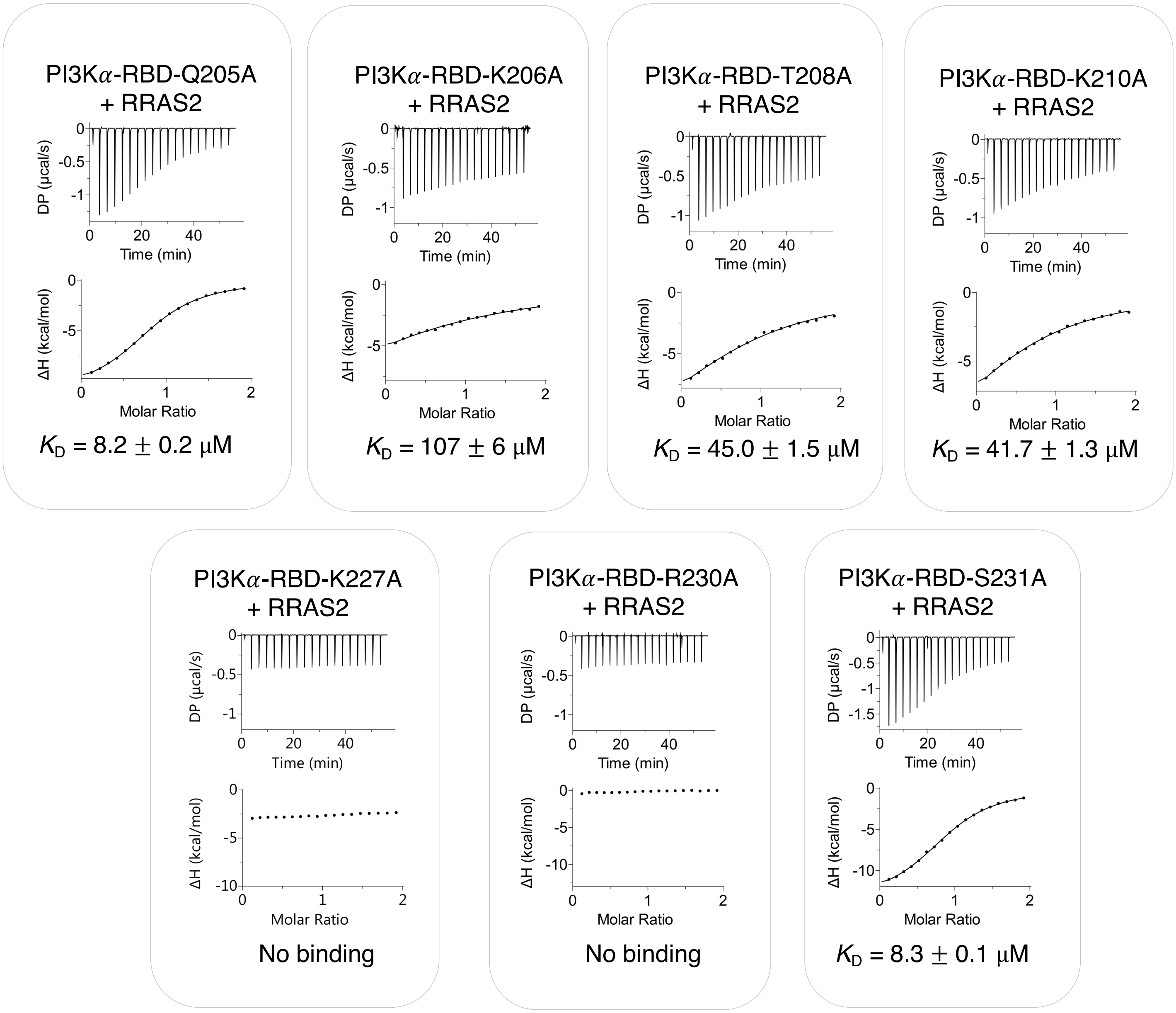

Supplementary Figure 12: ITC profiles showing the binding affinity of PI3Kα-RBD mutants with GMPPNP-bound RRAS2. PI3Kα-RBD residues present near the interaction interface were mutated to alanine and examined using ITC to assess their impact on the RRAS2-PI3Kα interaction. K_D values are presented as the mean ± standard deviation derived from two replicates.

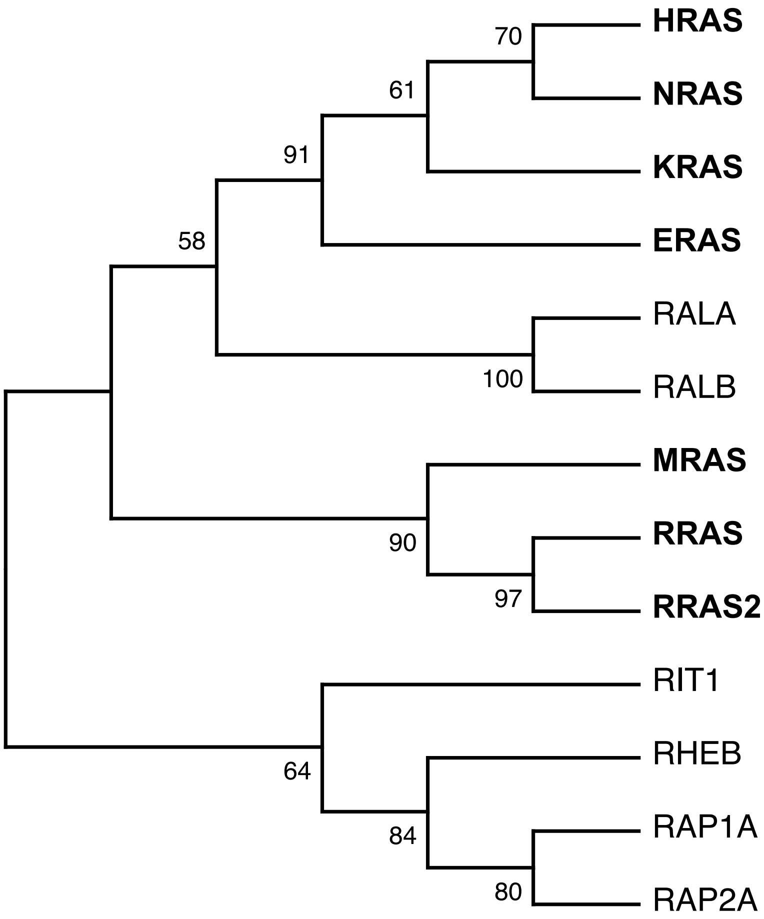

Supplementary Figure 13. Phylogenetic analysis of RAS family GTPases shown in Figure 4A. The tree with the highest log likelihood (~3102.01) is presented. Branches are annotated with the percentage of trees in which the associated taxa clustered together, reflecting the confidence in the clustering. RAS proteins that have been shown to bind to PI3Kα are shown in bold. UniProt IDs of the RAS isoforms used for this analysis are KRAS (P01116), HRAS (P01112), NRAS (P01111), RRAS (P10301), RRAS2 (P62070), MRAS (O14807), ERAS (Q7Z444), RALA (P11233), RALB (P11234), RAP1A (P62834), RAP2A (P10114), RIT1 (Q92963), and RHEB (Q15382).

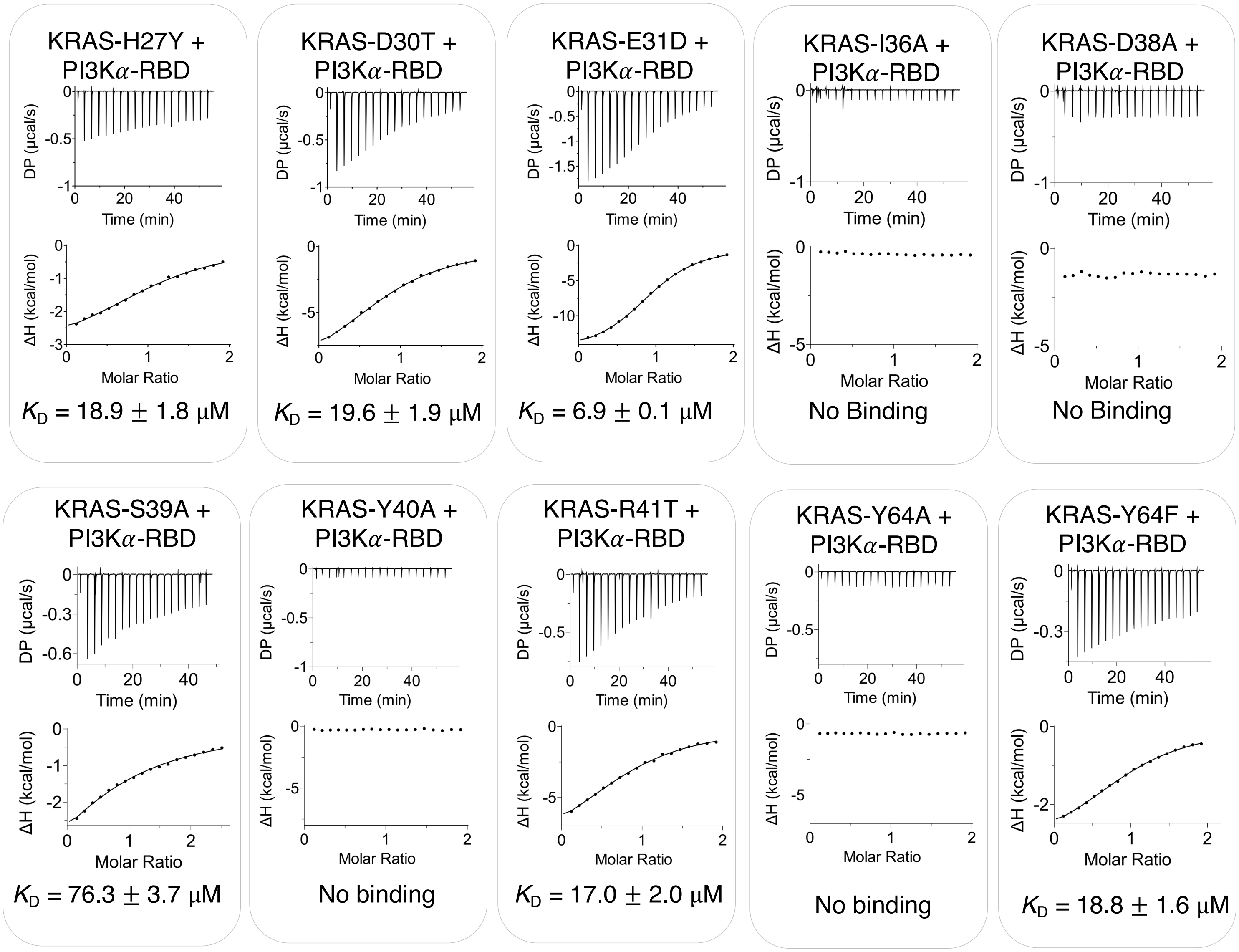

Supplementary Figure 14: ITC profiles illustrating the binding affinity of interface mutants of KRAS (GMPPNP-bound) with PI3Kα-RBD. KRAS interface residues were mutated to either alanine or the corresponding RRAS2 residues, and the binding affinities of these mutants with PI3Kα-RBD were assessed using ITC. K_D values are presented as the mean ± standard deviation from two replicates.

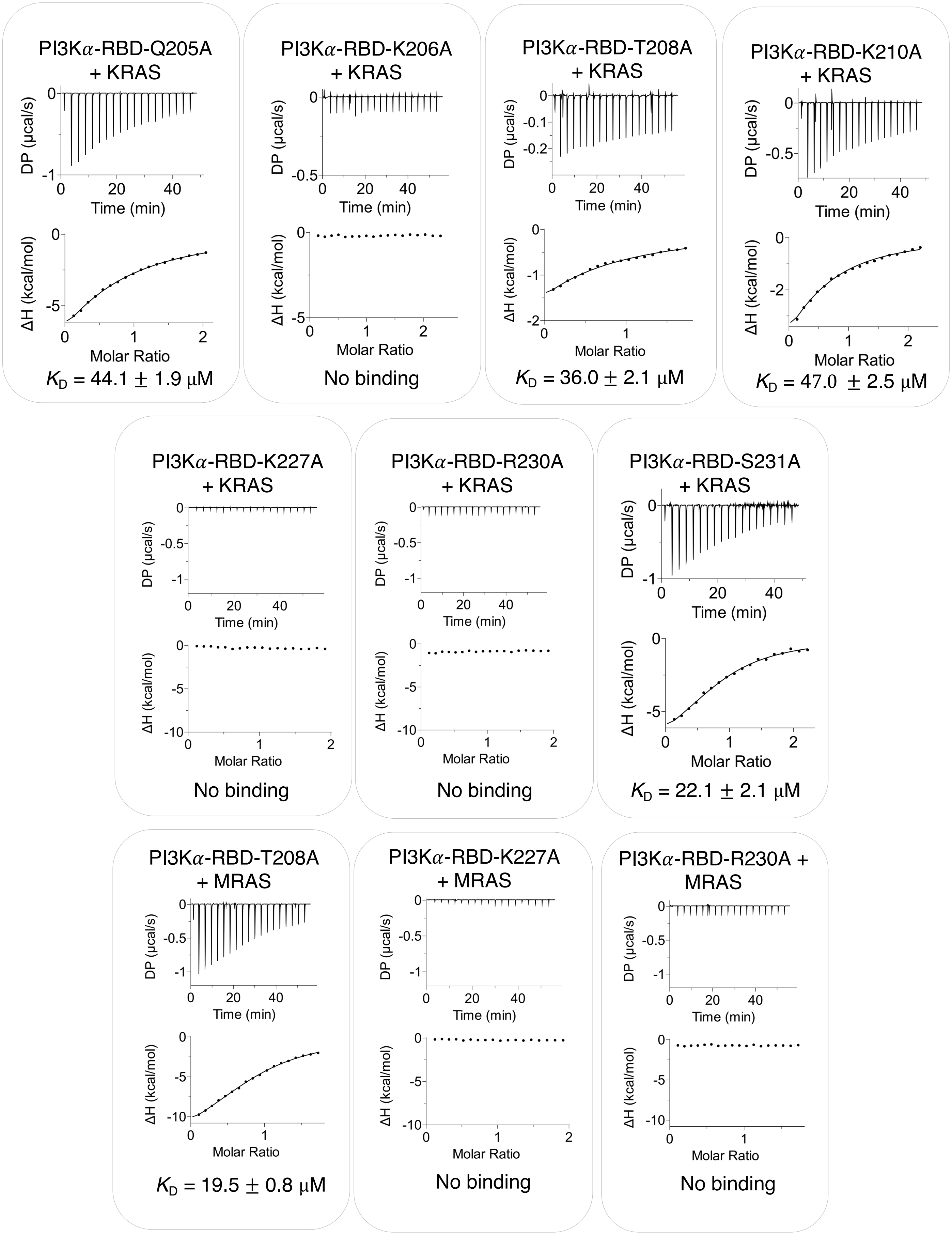

Supplementary Figure 15: ITC profiles showing the binding affinity of PI3Kα mutants with KRAS and MRAS. PI3Kα residues present near the interaction interface were mutated and examined using ITC to assess their impact on the interaction with GMPPNP-bound KRAS or MRAS. K_D values are presented as the mean ± standard deviation derived from two replicates.

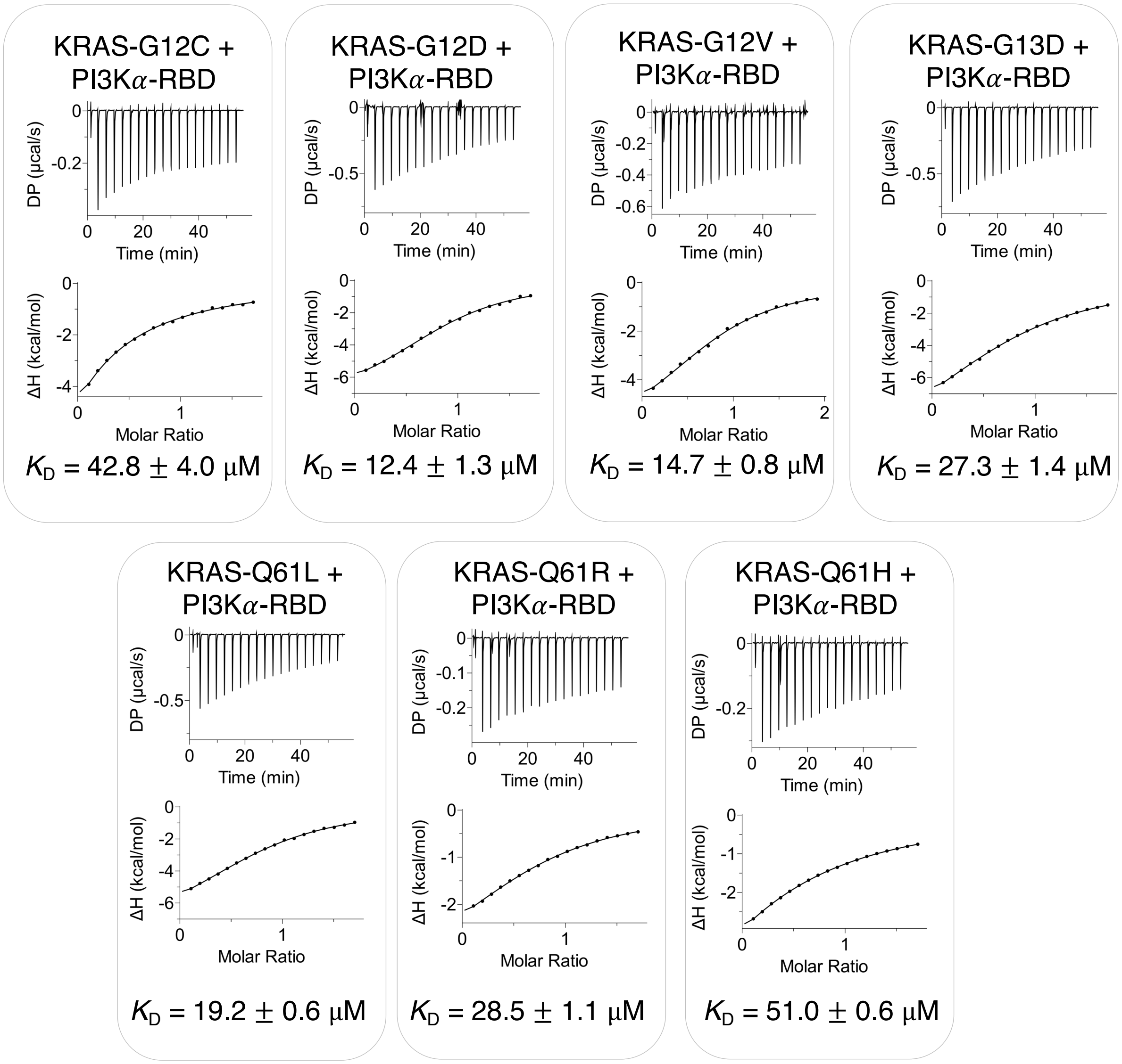

Supplementary Figure 16: ITC profiles illustrating the binding affinity of oncogenic mutants of KRAS with PI3Kα-RBD. The binding affinities of oncogenic KRAS mutants (GMPPNP-bound) at positions 12, 13, and 61 with PI3Kα-RBD were evaluated using ITC to determine their impact on the KRAS-PI3Kα interaction. K_D values are presented as the mean ± standard deviation from two replicates.

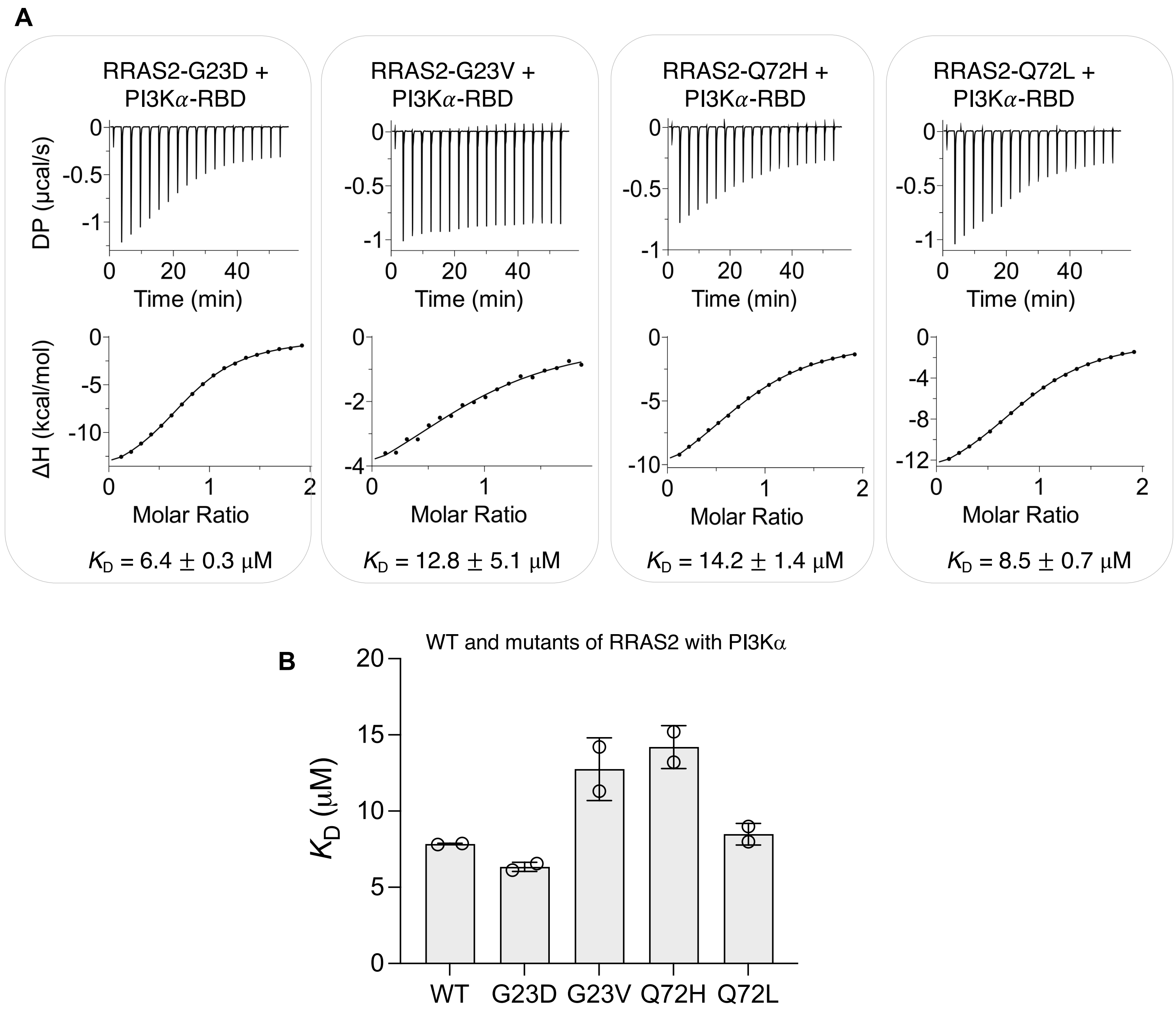

Supplementary Figure 17: ITC profiles showing the binding affinity of clinically relevant mutants of RRAS2 with PI3Kα-RBD. (A) Clinically relevant mutants (G23D, G23V, Q72H, and Q72L) of RRAS2 (GMPPNP-bound) were examined using ITC to assess their impact on the RRAS2-PI3Kα interaction. K_D values are presented as the mean ± standard deviation derived from two replicates. (**B**) A bar graph illustrating the binding affinity (K_D) of WT and mutants of RRAS2 with PI3Kα shown in panel A. The standard deviation is shown as error bars, with two replicates shown as circles.

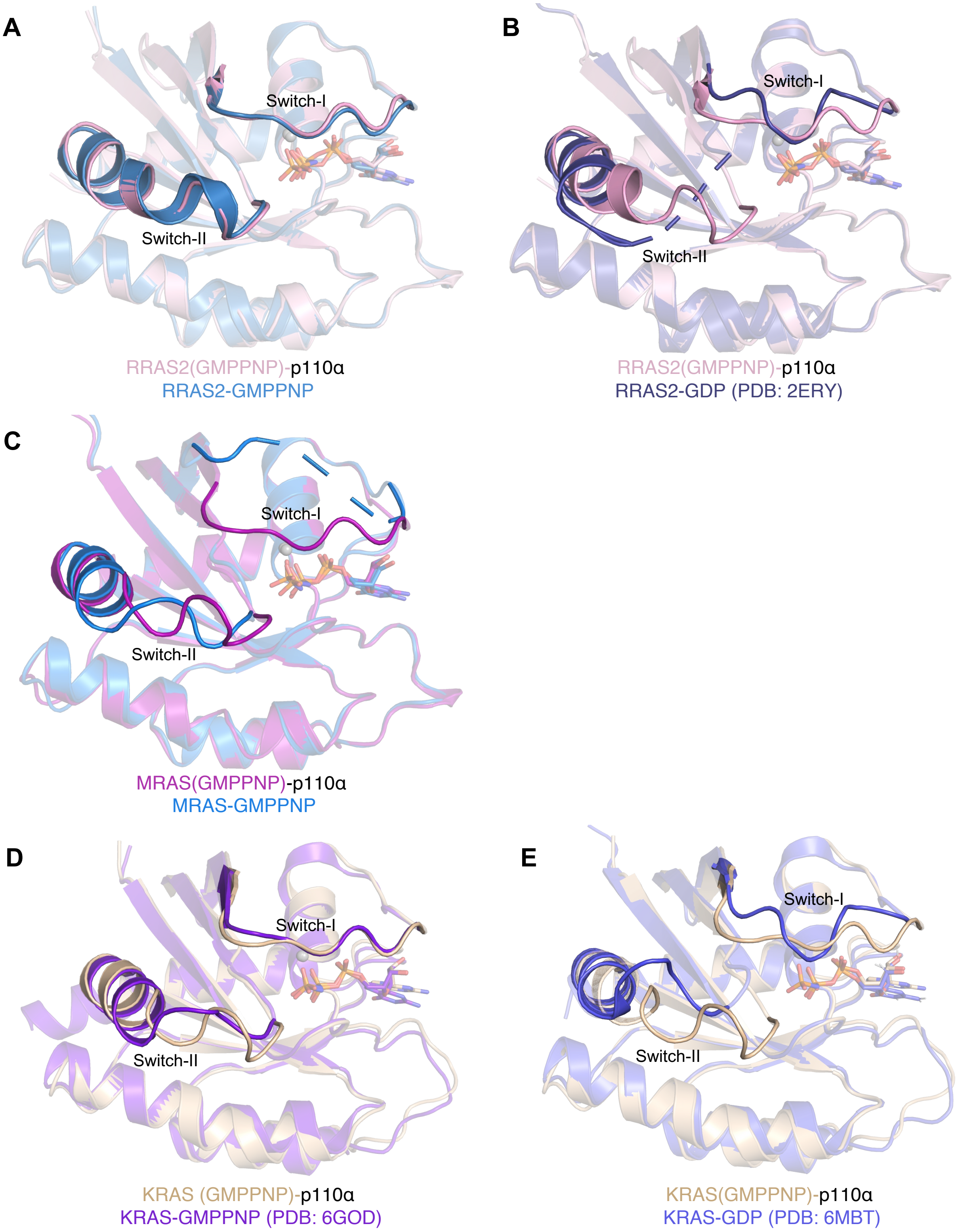

Supplementary Figure 18. Comparison of RRAS2, MRAS, and KRAS in free form and in complex with their respective p110α complexes to examine conformational changes in the switch regions upon p110α binding. (A, B) Structural superposition of the (A) GMPPNP-bound (navy blue) and (B) GDP-bound RRAS2 (blue) with RRAS2 (pink) in the RRAS2-p110α complex. **(C)** Structural superposition of GMPPNP-bound MRAS (marine blue) with MRAS (magenta) in the MRAS-p110α complex. **(D, E)** Structural superposition of the (D) GMPPNP-bound (purple) and (E) GDP-bound KRAS (dark blue) with KRAS (wheat) in the KRAS-p110α complex. For clarity, only the switch regions are highlighted in these panels.

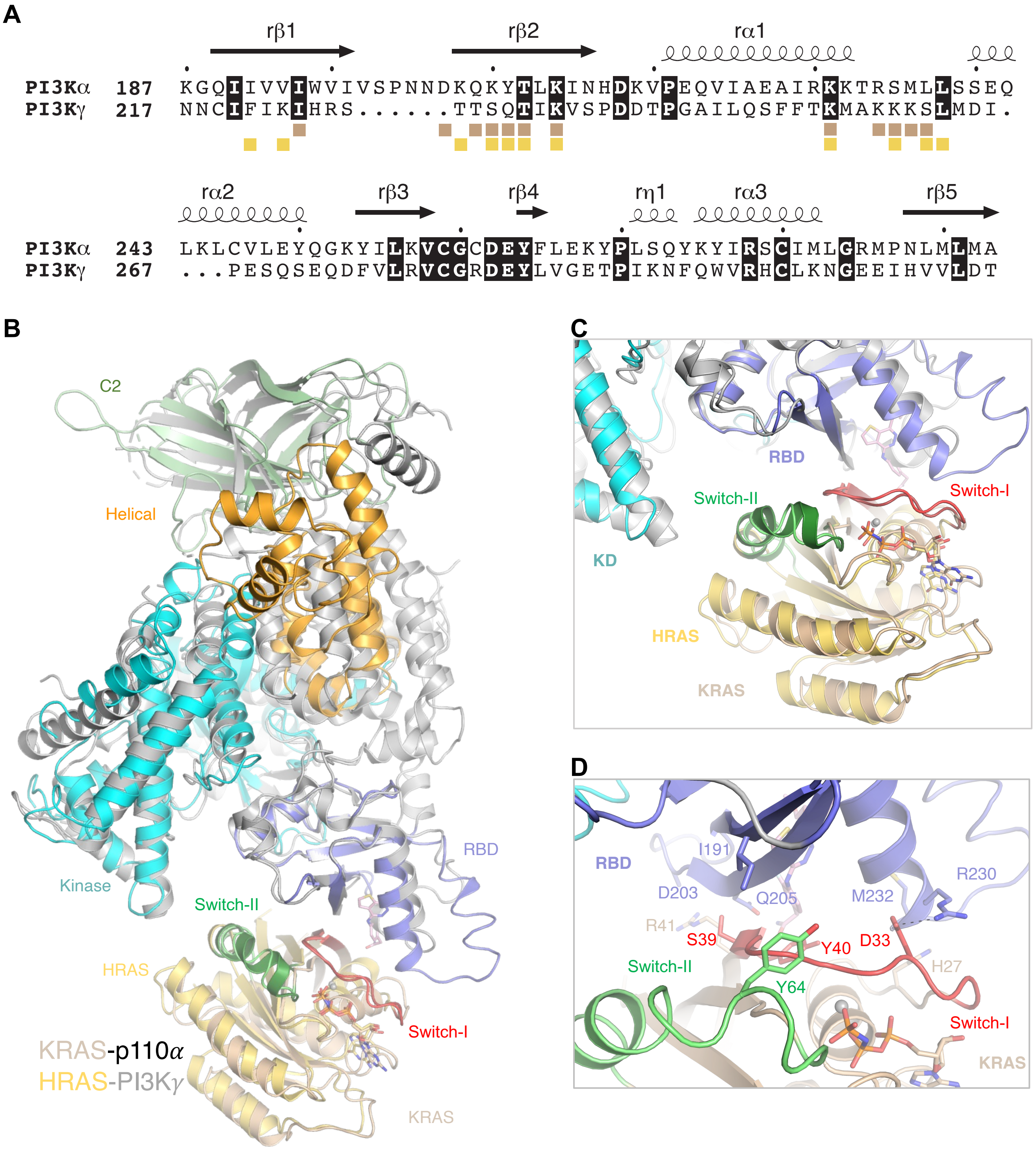

Supplementary Figure 19: Structural comparison of the KRAS-p110α complex with the HRAS-PI3Kγ complex. (A) Structure-based sequence alignment of the RBD region in p110α and PI3Kγ isoforms with fully conserved residues highlighted in black. Tan squares denote p110α residues engaging with KRAS, while yellow squares indicate PI3Kγ residues interacting with HRAS. **(B)** Overlay of the crystal structure of the KRAS-p110α complex with the previously reported HRAS-PI3Kγ complex (PDB: 1HE8), aligned using PI3K isoforms. **(C)** Enlarged view of the interaction interface from panel B, revealing differences between RAS proteins and the RBD of p110α and PI3Kγ. **(D)** A detailed view of the switch regions of KRAS with interactions unique to the KRAS-p110α complex, as shown in panel A.

Supplementary Table 1: The thermodynamic parameters from ITC experiments measuring binding affinity between GMPPNP-bound RAS GTPases and four class I PI3K isoforms. Thermodynamic parameters: K_D (Dissociation Constant), ΔH (Enthalpy Change), −TΔS (Entropy Contribution to Free Energy), ΔG (Gibbs Free Energy), N-value (Stoichiometry) and SD (Standard deviation). In these ITC experiments, a lower ΔH indicates stronger, more favorable interactions, while a higher ΔH suggests weaker binding. A higher ΔS reflects increased disorder and more favorable binding, whereas a lower ΔS suggests less favorable entropic contributions. A lower ΔG signifies a stronger, spontaneous interaction, while a higher ΔG indicates weaker binding. Lower K_D values correspond to higher binding affinity, whereas higher K_D values suggest weaker interactions. The N-value represents the stoichiometry of the interaction.

Protein (Syringe)	Protein (Cell)	K_D (mean ± SD) (μM)	ΔH ± SD (kcal mol^{-1})	-TΔS ± SD (kcal mol^{-1})	ΔG ± SD (kcal mol^{-1})	N ± SD (sites)	Replicates
RRAS2	PI3Kα	3.9 ± 0.9	-16.6 ± 1.8	9.2 ± 1.9	-7.4 ± 0.1	0.80 ± 0.21	2
RRAS2	PI3Kβ	No binding					2
RRAS2	PI3Kγ	5.5 ± 0.5	-12.0 ± 0.7	4.8 ± 0.8	-7.2 ± 0.1	0.90 ± 0.02	2
RRAS2	PI3Kδ	13.8 ± 1.2	-14.8 ± 0.7	8.2 ± 0.7	-6.6 ± 0.1	0.65 ± 0.03	2
KRAS4b	PI3Kα	16.9 ± 0.8	-3.1 ± 0.1	-3.4 ± 0.1	-6.5 ± 0.1	0.99 ± 0.01	2
* KRAS4b	PI3Kα	4.6 ± 0.4	-7.8 ± 0.1	0.5 ± 0.1	-7.3 ± 0.1	0.63 ± 0.02	2
KRAS4b	PI3Kβ	No binding					2
KRAS4b	PI3Kγ	20.3 ± 0.3	-14.1 ± 0.4	7.7 ± 0.4	-6.4 ± 0.1	0.80 ± 0.22	2
KRAS4b	PI3Kδ	13.5 ± 1.2	-10.9 ± 0.9	4.3 ± 0.9	-6.7 ± 0.1	0.61 ± 0.21	2
HRAS	PI3Kα	25.3 ± 0.2	-8.4 ± 0.6	2.1 ± 0.7	-6.3 ± 0.1	1.02 ± 0.02	2
NRAS	PI3Kα	18.5 ± 0.2	-7.9 ± 0.6	1.8 ± 0.6	-6.1 ± 0.1	0.98 ± 0.02	2
RRAS	PI3Kα	27.6 ± 1.9	-6.8 ± 0.3	0.6 ± 0.4	-6.2 ± 0.1	1.05 ± 0.03	2
MRAS	PI3Kα	5.3 ± 0.7	-16.9 ± 0.3	9.7 ± 0.2	-7.2 ± 0.1	0.95 ± 0.04	2
RIT1	PI3Kα	No binding					2
RRAS2	PI3Kα-RBD	7.8 ± 0.1	-18.3 ± 0.1	11.3 ± 0.1	-6.9 ± 0.1	0.79 ± 0.01	2
KRAS4b	PI3Kα-RBD	23.1 ± 1.3	-8.1 ± 0.3	1.8 ± 0.4	-6.3 ± 0.1	0.92 ± 0.02	2
HRAS	PI3Kα-RBD	29.1 ± 0.2	-9.3 ± 0.2	3.1 ± 0.2	-6.2 ± 0.1	0.94 ± 0.02	2
NRAS	PI3Kα-RBD	24.2 ± 1.5	-9.3 ± 0.7	1.2 ± 0.2	-6.1 ± 0.1	0.80 ± 0.12	2
RRAS	PI3Kα-RBD	36.4 ± 0.3	-7.3 ± 0.2	2.9 ± 0.7	-6.3 ± 0.1	1.07 ± 0.05	2
MRAS	PI3Kα-RBD	5.5 ± 0.3	-16.8 ± 0.2	9.6 ± 1.5	-7.2 ± 0.1	0.81 ± 0.11	2
RIT1	PI3Kα-RBD	No binding					

* ITC experiment performed at 50 mM NaCl instead of 150 mM NaCl.

Supplementary Table 2: **The thermodynamic parameters from ITC experiments measuring binding affinity between GMPPNP-bound RRAS2/MRAS and PI3Kα/ PI3Kα–RBD.**

Protein (Syringe)	Protein (Cell)	K_D (mean ± SD) (μM)	ΔH ± SD (kcal mol^{-1})	-TΔS ± SD (kcal mol^{-1})	ΔG ± SD (kcal mol^{-1})	N ± SD (sites)	Replicates
**RRAS2 with PI3Kα**							
RRAS2-Q36A	PI3Kα	2.1 ± 0.6	-15.0 ± 0.3	7.3 ± 0.4	-7.8 ± 0.2	0.95 ± 0.02	2
RRAS2-G23D	PI3Kα-RBD	6.4 ± 0.3	-16.0 ± 0.7	8.9 ± 0.7	-7.1 ± 0.1	0.75 ± 0.05	2
RRAS2-G23V	PI3Kα-RBD	12.8 ± 5.1	-6.9 ± 1.8	2.6 ± 1.7	-6.7 ± 0.1	0.91 ± 0.11	2
RRAS2-Q36A	PI3Kα-RBD	4.9 ± 0.5	-11.8 ± 0.6	4.5 ± 0.6	-7.2 ± 0.1	1.12 ± 0.11	2
RRAS2-Y38F	PI3Kα-RBD	4.3 ± 0.5	-15.5 ± 1.6	8.1 ± 1.6	-7.3 ± 0.1	0.92 ± 0.10	2
RRAS2-D42E	PI3Kα-RBD	16.1 ± 4.3	-3.9 ± 0.3	-2.7 ± 0.5	-6.6 ± 0.2	0.89 ± 0.04	2
RRAS2-D42A	PI3Kα-RBD	66.9 ± 8.1	-4.7 ± 0.3	-9.7 ± 0.3	-5.7 ± 0.1	1.20 ± 0.12	2
RRAS2-D44A	PI3Kα-RBD	28.2 ± 0.6	-13.4 ± 2.1	7.2 ± 2.0	-6.2 ± 0.1	0.81 ± 0.10	2
RRAS2-E48A	PI3Kα-RBD	32.4 ± 0.4	-2.4 ± 0.6	-3.8 ± 0.6	-6.1 ± 0.1	0.92 ± 0.11	2
RRAS2-D49A	PI3Kα-RBD	No binding					2
RRAS2-Y51A	PI3Kα-RBD	No binding					2
RRAS2-T52R	PI3Kα-RBD	28.5 ± 2.3	-15.7 ± 4.0	9.5 ± 4.0	-6.2 ± 0.1	1.01 ± 0.02	2
RRAS2-R63A	PI3Kα-RBD	6.4 ± 0.7	-16.7 ± 0.9	9.6 ± 0.9	-7.1 ± 0.1	0.90 ± 0.12	2
RRAS2-Q72H	PI3Kα-RBD	14.2 ± 1.4	-13.9 ± 0.8	7.2 ± 0.8	-6.6 ± 0.1	0.85 ± 0.01	2
RRAS2-Q72L	PI3Kα-RBD	8.5 ± 0.7	-15.2 ± 0.2	8.2 ± 0.3	-6.9 ± 0.1	0.88 ± 0.04	2
RRAS2-E74A	PI3Kα-RBD	12.4 ± 1.5	-13.3 ± 0.2	6.6 ± 0.3	-6.7 ± 0.1	0.83 ± 0.02	2
RRAS2-F75A	PI3Kα-RBD	21.5 ± 1.7	-2.9 ± 0.1	-3.5 ± 0.2	-6.4 ± 0.1	1.11 ± 0.12	2
RRAS2-M78A	PI3Kα-RBD	134 ± 25	-9.2 ± 0.5	3.9 ± 0.6	-5.3 ± 0.1	1.20 ± 0.21	2
RRAS2	PI3Kα-RBD-Q205A	8.2 ± 0.2	-11.2 ± 0.1	4.2 ± 0.1	-6.9 ± 0.1	0.86 ± 0.01	2
RRAS2	PI3Kα-RBD-K206A	107 ± 6	-16.4 ± 1.6	10.9 ± 1.5	-5.4 ± 0.1	0.92 ± 0.10	2
RRAS2	PI3Kα-RBD-T208A	45.0 ± 1.5	-15.1 ± 0.8	9.2 ± 0.8	-5.9 ± 0.1	0.90 ± 0.11	2
RRAS2	PI3Kα-RBD-K210A	41.7 ± 1.3	-12.8 ± 1.8	6.8 ± 1.8	-5.9 ± 0.1	0.81 ± 0.13	2
RRAS2	PI3Kα-RBD-K227A	No binding					2
RRAS2	PI3Kα-RBD-R230A	No binding					2
RRAS2	PI3Kα-RBD-S231A	8.3 ± 0.1	-13.8 ± 0.1	6.8 ± 0.1	-6.9 ± 0.1	0.88 ± 0.05	2
**MRAS with PI3Kα**							
MRAS-Q35A	PI3Kα	5.1 ± 0.8	-12.7 ± 0.3	5.5 ± 0.4	-7.2 ± 0.1	0.96 ± 0.01	2
MRAS-Q35A	PI3Kα-RBD	5.3 ± 0.1	-12.8 ± 0.4	5.6 ± 0.4	-7.2 ± 0.1	0.87 ± 0.03	2
MRAS	PI3Kα-RBD-T208A	19.5 ± 0.8	-14.6 ± 0.1	8.2 ± 0.1	-6.4 ± 0.1	0.93 ± 0.05	2
MRAS	PI3Kα-RBD-K227A	No binding					2
MRAS	PI3Kα-RBD-R230A	No binding					2

Supplementary Table 3: The thermodynamic parameters from ITC experiments measuring binding affinity between GMPPNP-bound KRAS and PI3Kα/PI3Kα–RBD.

Protein (Syringe)	Protein (Cell)	K_D (mean ± SD) (μM)	ΔH ± SD (kcal mol^{-1})	-TΔS ± SD (kcal mol^{-1})	ΔG ± SD (kcal mol^{-1})	N ± SD (sites)	Replicates
**KRAS with PI3Kα**							
KRAS+D927	PI3Kα	0.036 ± 0.010	-13.9 ± 2.9	3.7 ± 2.7	-10.2 ± 0.1	0.73 ± 0.03	2
KRAS-D927	PI3Kα-RBD	0.049 ± 0.006	-23.9 ± 0.3	13.9 ± 0.2	-9.9 ± 0.1	0.98 ± 0.05	2
KRAS-Q25A	PI3Kα	4.9 ± 0.9	-10.5 ± 0.4	3.3 ± 0.5	-7.3 ± 0.1	0.91 ± 0.01	2
KRAS-G12C	PI3Kα-RBD	42.8 ± 4.0	-14.0 ± 0.4	8.0 ± 0.4	-5.9 ± 0.1	0.36 ± 0.01*	2
KRAS-G12D	PI3Kα-RBD	12.4 ± 1.3	-7.4 ± 0.4	7.2 ± 0.4	-6.7 ± 0.1	0.91 ± 0.12	2
KRAS-G12V	PI3Kα-RBD	14.7 ± 0.8	-6.7 ± 0.1	0.1 ± 0.1	-6.6 ± 0.1	0.72 ± 0.20*	2
KRAS-G13D	PI3Kα-RBD	27.3 ± 1.4	-11.5 ± 0.1	5.3 ± 0.1	-6.2 ± 0.1	0.81 ± 0.06	2
KRAS-Q25A	PI3Kα-RBD	5.7 ± 0.2	-10.5 ± 0.1	3.3 ± 0.1	-7.2 ± 0.1	0.91 ± 0.01	2
KRAS-H27Y	PI3Kα-RBD	18.9 ± 1.8	-3.1 ± 0.3	-3.4 ± 0.4	-6.5 ± 0.1	1.16 ± 0.03	2
KRAS-D30T	PI3Kα-RBD	19.6 ± 1.9	-10.4 ± 0.4	3.9 ± 0.5	-6.4 ± 0.1	0.89 ± 0.01	2
KRAS-E31D	PI3Kα-RBD	6.9 ± 0.1	-15.0 ± 0.7	7.9 ± 0.7	-7.0 ± 0.1	1.05 ± 0.02	2
KRAS-I36A	PI3Kα-RBD	No binding					2
KRAS-D38A	PI3Kα-RBD	No binding					2
KRAS-S39A	PI3Kα-RBD	76.3 ± 3.7	-7.8 ± 2.2	2.2 ± 2.2	-5.6 ± 0.1	0.63 ± 0.30	2
KRAS-Y40A	PI3Kα-RBD	No binding					2
KRAS-R41T	PI3Kα-RBD	17.0 ± 2.0	-8.0 ± 1.9	1.5 ± 2.1	-6.5 ± 0.3	0.86 ± 0.03	2
KRAS-Q61H	PI3Kα-RBD	51.0 ± 0.6	-9.7 ± 3.1	3.9 ± 3.1	-5.9 ± 0.1	0.60 ± 0.31*	2
KRAS-Q61L	PI3Kα-RBD	19.2 ± 0.6	-7.7 ± 0.6	1.2 ± 0.7	-6.4 ± 0.1	0.91 ± 0.10	2
KRAS-Q61R	PI3Kα-RBD	28.5 ± 1.1	-4.1 ± 0.4	-2.1 ± 0.4	-6.2 ± 0.1	0.77 ± 0.03	2
KRAS-Y64A	PI3Kα-RBD	No binding					2
KRAS-Y64F	PI3Kα-RBD	18.8 ± 1.6	-3.1 ± 0.2	-3.4 ± 0.1	-6.5 ± 0.1	1.11 ± 0.10	2
KRAS	PI3Kα-RBD-V193K	23.2 ± 1.6	-4.3 ± 1.3	-2.1 ± 1.2	-6.3 ± 0.1	0.82 ± 0.21	2
KRAS	PI3Kα-RBD-Q205A	44.1 ± 1.9	-11.3 ± 2.8	5.4 ± 2.8	-5.9 ± 0.1	0.81 ± 0.22	2
KRAS	PI3Kα-RBD-K206A	No binding					2
KRAS	PI3Kα-RBD-T208A	36.0 ± 2.1	-4.9 ± 0.8	-1.2 ± 0.7	-6.1 ± 0.1	0.55 ± 0.32*	2
KRAS	PI3Kα-RBD-K210A	47.0 ± 2.5	-6.6 ± 4.5	3.2 ± 1.0	-5.9 ± 0.1	0.43 ± 0.31*	2
KRAS	PI3Kα-RBD-K227A	No binding					2
KRAS	PI3Kα-RBD-R230A	No binding					2
KRAS	PI3Kα-RBD-S231A	22.2 ± 2.1	-7.7 ± 0.6	1.3 ± 0.6	-6.4 ± 0.1	0.87 ± 0.02	2

* These mutants exhibited precipitation during the ITC experiment, rendering the N-value less reliable due to its dependence on accurate protein concentration, unlike other thermodynamic parameters.

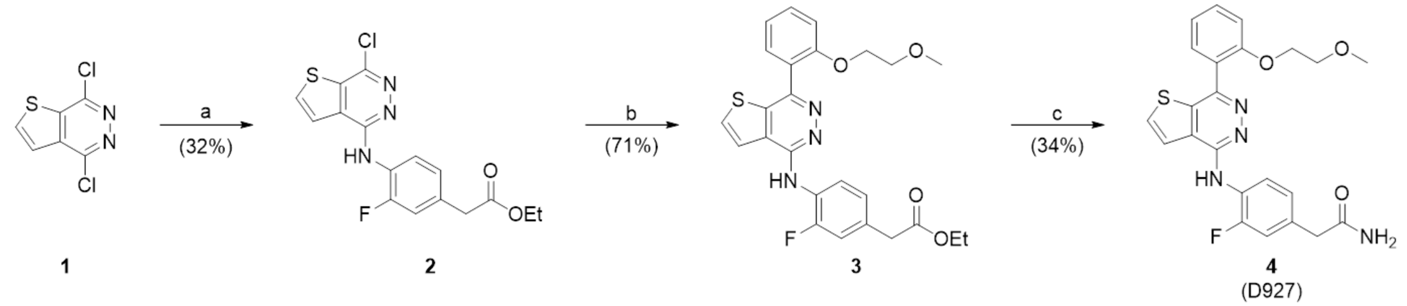

Scheme 1. Reagents and conditions: (a) 4-amino-3-fluorophenylacetic acid, EtOH, MW 120 °C, 1 hr; (b) 2-(2-methoxyethoxy)phenylboronic acid, $Pd(PPh_3)_4$, Cs_2CO_3, Dioxane, MW 90 °C, 30 min; (c) 7N Ammonia in MeOH, MW 130 °C, 2h.
